# Supplementary material for: Screen Time and Its Association with Vegetables, Fruits, Snacks and Sugary Sweetened Beverages Intake among Chinese Preschool Children in Changsha, Hunan Province: A Cross-Sectional Study
Source: Nutrients. 2022 Oct 1;14(19):4086. doi: 10.3390/nu14194086 (PMC9572133; doi:10.3390/nu14194086)
Supplement: Supplementary file 1 [file nutrients-14-04086-s001.zip › nutrients-1929698-supplementary.pdf]

Screen time and its association with vegetables, fruits, snacks and sugar-sweetened beverages intake among Chinese preschool children in Changsha, Hunan Province: A Cross-Sectional study.

Supplementary Materials:

Table S1 The characteristics of Children Eating Behaviors by screen time, vegetables, fruits, SSBs and snacks (n = 1567, n (%))

Variables	Total	Screen Time		Vegetables		Fruits		Snacks		SSBs	
		⩾ 1h	***P***	< once/day	***P***	< once/day	***P***	> once/week	***P***	> once/week	***P***
	(n=1567)	851 (54.3)		558 (35.6)		665 (42.4)		1313 (83.8)		362 (23.1)	
**Food Responsiveness**			**0.009**		0.430		0.075		0.265		**0.014**
Low	792 (50.5)	404 (47.5)		290 (52.0)		354 (53.2)		655 (49.9)		162 (44.8)	
High	775 (49.5)	447 (52.5)		268 (48.0)		311 (46.8)		658 (50.1)		200 (55.2)	
**Enjoyment of Food**			0.204		**<0.001**		**<0.001**		**0.035**		0.335
Low	971 (62.0)	540 (63.5)		389 (69.7)		452 (68.0)		829 (63.1)		216 (59.7)	
High	596 (38.0)	311 (36.5)		169 (30.3)		213 (32.0)		484 (36.9)		146 (40.3)	
**Satiety Responsiveness**			0.798		**<0.001**		**<0.001**		**<0.001**		0.897
Low	812 (51.8)	444 (52.2)		255 (45.7)		309 (46.5)		653 (49.7)		186 (51.4)	
High	755 (48.2)	407 (47.8)		303 (54.3)		356 (53.5)		660 (50.3)		176 (48.6)	
**Slowness in Eating**			0.115		0.347		**0.019**		0.068		0.054
Low	947 (60.4)	530 (62.3)		328 (58.8)		379 (57.0)		780 (59.4)		235 (64.9)	
High	620 (39.6)	321 (37.7)		230 (41.2)		286 (43.0)		533 (40.6)		127 (35.1)	
**Emotional Over-eating**			**0.001**		0.945		0.419		0.595		**0.001**
Low	812 (51.8)	409 (48.1)		288 (51.6)		353 (53.1)		676 (51.5)		160 (44.2)	
High	755 (48.2)	442 (51.9)		270 (48.4)		312 (46.9)		637 (48.5)		202 (55.8)	
**Emotional Under-eating**			0.719		0.723		0.848		**0.007**		0.472
Low	1108 (70.7)	598 (70.3)		391 (70.1)		468 (70.4)		910 (69.3)		250 (69.1)	
High	459 (29.3)	253 (29.7)		167 (29.9)		197 (29.6)		403 (30.7)		112 (30.9)	

**Desire to Drink**			0.776		**0.019**		0.123		0.053		**<0.001**
Low	980 (62.5)	529 (62.2)		371 (66.5)		431 (64.8)		807 (61.5)		183 (50.6)	
High	587 (37.5)	322 (37.8)		187 (33.5)		234 (35.2)		506 (38.5)		179 (49.4)	
**Food Fussiness**			**0.015**		**<0.001**		**<0.001**		0.324		**0.031**
Low	630 (40.2)	317 (37.3)		162 (29.0)		232 (34.9)		518 (39.5)		147 (40.6)	
Medium	506 (32.3)	298 (35.0)		183 (32.8)		214 (32.2)		426 (32.4)		133 (36.7)	
High	431 (27.5)	236 (27.7)		213 (38.2)		219 (32.9)		369 (28.1)		82 (22.7)	
**Food Neophobia**			0.125		**<0.001**		**0.011**		0.575		0.256
Low	947 (60.4)	499 (58.6)		303 (54.3)		377 (56.7)		789 (60.1)		209 (57.7)	
High	620 (39.6)	352 (41.4)		255 (45.7)		288 (43.3)		524 (39.9)		153 (42.3)	

Compared by chi-square test. Bolding indicates statistically significant values, $P < 0.05$.

Table S2 The characteristics of Children Feeding Practice by screen time, vegetables, fruits, SSBs and snacks (n = 1567, n (%))

Variables	Total	**Screen Time** ⩾ **1h** 851 (54.3)	***P***	**Vegetables** **< once/day** 558 (35.6)	***P***	**Fruits** **< once/day** 665 (42.4)	***P***	**Snacks** **> once/week** 1313 (83.8)	***P***	**SSBs** **> once/week** 362 (23.1)	***P***
Perceived Responsibility			0.539		0.928		0.926		0.362		**0.002**
Low	894 (57.1)	492 (57.8)		317 (56.8)		378 (56.8)		742 (56.5)		378 (56.8)	
High	673 (42.9)	359 (42.2)		241 (43.2)		287 (43.2)		571 (43.5)		287 (43.2)	
Perceive Parent Weight			0.440		0.494		0.633		0.770		0.760
Low	1389 (88.6)	749 (88.0)		490 (87.8)		586 (88.1)		1162 (88.5)		586 (88.1)	
High	178 (11.4)	102 (12.0)		68 (12.2)		79 (11.9)		151 (11.5)		79 (11.9)	
Perceive Child Weight			0.217		0.137		0.154		0.131		0.444
Low	1346 (85.9)	722 (84.8)		469 (84.1)		561 (84.4)		1136 (86.5)		561 (84.4)	
High	221 (14.1)	129 (15.2)		89 (15.9)		104 (15.6)		177 (13.5)		104 (15.6)	
Concern			**0.008**		0.817		0.459		0.609		**<0.001**

Low	717 (45.8)	363 (42.7)		258 (46.2)		312 (46.9)		605 (46.1)		312 (46.9)	
High	850 (54.2)	488 (57.3)		300 (53.8)		353 (53.1)		708 (53.9)		353 (53.1)	
Restriction			**0.039**		0.116		0.931		**<0.001**		**<0.001**
Low	864 (55.1)	490 (57.6)		323 (57.9)		368 (55.3)		752 (57.3)		368 (55.3)	
High	703 (44.9)	361 (42.4)		235 (42.1)		297 (44.7)		561 (42.7)		297 (44.7)	
Food as Reward			0.579		0.875		0.370		**0.004**		0.055
Low	1135 (72.4)	611 (71.8)		406 (72.8)		490 (73.7)		932 (71.0)		490 (73.7)	
High	432 (27.6)	240 (28.2)		152 (27.2)		175 (26.3)		381 (29.0)		175 (26.3)	
Pressure to Eat			0.354		0.209		0.979		0.898		0.090
Low	929 (59.3)	514 (60.4)		343 (61.5)		395 (59.4)		777 (59.2)		395 (59.4)	
High	638 (40.7)	337 (39.6)		215 (38.5)		270 (40.6)		536 (40.8)		270 (40.6)	
Monitoring			**<0.001**		**<0.001**		**<0.001**		**<0.001**		**<0.001**
Low	1038 (66.2)	608 (71.4)		406 (72.8)		475 (71.4)		906 (69.0)		475 (71.4)	
High	529 (33.8)	243 (28.6)		152 (27.2)		190 (28.6)		407 (31.0)		190 (28.6)	

Compared by chi-square test. Bolding indicates statistically significant values, $P < 0.05$.
